# Injectable platelet-rich fibrin with demineralized freeze-dried bone allograft compared to demineralized freeze-dried bone allograft in intrabony defects of patients with stage-III periodontitis: a randomized controlled clinical trial

**DOI:** 10.1007/s00784-023-04954-y

**Published:** 2023-03-31

**Authors:** Mashaal Mohammed Alshoiby, Karim Mohamed Fawzy El-Sayed, Weam Elbattawy, Manal Mohamed Hosny

**Affiliations:** 1 Oral Medicine and Periodontology Department, Faculty of Dentistry, Cairo University, Al Saraya Str. 11, Manial, Cairo, Egypt; 2 Clinic for Conservative Dentistry and Periodontology, School of Dental Medicine, Christian Albrechts University, Kiel, Germany; 3 Stem Cells and Tissue Engineering Research Unit, Faculty of Dentistry, Cairo University, Cairo, Egypt

**Keywords:** Platelet rich-fibrin, Allograft, Periodontal, Regeneration, Intrabony, Periodontitis

## Abstract

**Aim:**

The current randomized controlled clinical trial assessed the effect of injectable platelet-rich fibrin (I-PRF) combined with demineralized freeze-dried bone allograft (DFDBA) compared to DFDBA alone in the management of intrabony defects of stage-III periodontitis patients.

**Methodology:**

Following sample size calculation, twenty stage-III periodontitis patients with ≥ 5 mm clinical attachment level (CAL)-loss and ≥ 3 mm intrabony defects were randomized into test (I-PRF + DFDBA; *n* = 10) and control (DFDBA; *n* = 10) groups. CAL (primary outcome), periodontal probing depth (PPD), gingival recession depth (GRD), full-mouth plaque scores (FMPS), full-mouth bleeding scores (FMBS), radiographic linear defect depth (RLDD), and bone fill (secondary outcomes) were examined at baseline, 3, 6, and 9 months post-surgically.

**Results:**

I-PRF + DFDBA and DFDBA independently demonstrated significant intragroup CAL-gain, PPD-, and RLDD-reduction at 3, 6, and 9 months (*p* < 0.05), with no significant intergroup differences observed (*p* > 0.05). CAL-gain (mean ± SD) of 2.40 ± 0.70 mm and 2.50 ± 0.85 mm and PPD-reduction of 3.50 ± 1.18 mm and 2.80 ± 0.42 mm were demonstrated for I-PRF + DFDBA and DFDBA at 9 months respectively. Both groups showed significant intragroup RLDD improvement, with a RLDD of 3.58 ± 0.66 mm and 3.89 ± 1.57 mm for I-PRF + DFDBA and DFDBA at 9 months respectively. Stepwise linear regression analysis revealed that baseline RLDD and bone fill at 9 months were significant predictors of CAL (*p* < 0.05).

**Conclusion:**

Within the present study’s limitations, DFDBA with or without I-PRF resulted in significant improvement in clinical and radiographic periodontal parameters in the surgical treatment of periodontal intrabony defects of stage-III periodontitis patients. Addition of I-PRF to DFDBA does not appear to significantly enhance the DFDBA’s reparative/regenerative outcomes.

**Clinical relevance:**

Within the current study’s limitations, routinely adding I-PRF to DFDBA cannot be recommended to significantly improve DFDBA’s treatment outcomes in intrabony defects.

## Introduction

Periodontitis is a chronic inflammatory disorder, associated with dysbiotic plaque biofilms, resulting untreated in progressive destruction of the tooth-supporting apparatus and intrabony periodontal defects [1]. Such defects present risk factors for further disease progression, and their therapy improves teeth prognosis [2]. In this context, a number of periodontal approaches were advocated, employing barrier membranes, enamel matrix derivatives, bone grafts, or growth factor concentrates [3].

The osteoinductive demineralized freeze-dried bone allograft (DFDBA) harbors a variety of growth/differentiation factors, notably bone morphogenetic proteins (BMPs) 2, 4, and 7, and is inferred to promote periodontal repair/regeneration, with significant PPD-reduction, CAL-gain, and bone fill [4]. Injectable platelet-rich fibrin (I-PRF), a liquid autologous platelet concentrate introduced based on the “low-speed centrifugation concept” [5, 6], further harbors a variety of growth/differentiation factors (GFs), with reported positive attributes on angiogenesis, inflammation, and periodontal wound healing [6–8]. Recently, it was proposed that mixing I-PRF with bone grafts, forming a gelatinous fibrin-graft-amalgamate rich in growth/differentiation factors (sticky bone), enhanced the graft’s biological properties, handling, and stability [9]. Through the I-PRF-contained growth/differentiation factors in addition to its fibrin meshwork, “sticky bone” was proposed to endorse periodontal healing processes, enhancing periodontal cell adhesion, osteoprogenitor cell selection, osteoblastic cell viability, attachment, proliferation, and differentiation [9, 10] as well as bone regeneration, while decreasing epithelial soft tissue ingrowth into periodontal intrabony defects [11, 12]. In addition to its enhanced handling characteristics, the clinically improved adaptation and stabilization properties of “sticky bone” are believed to prevent micro- and macro-mobility of the graft introduced into the periodontal defects, with enhanced wound healing and regeneration attributes [13].

Still, limited data is available on the clinical potential of I-PRF in combination with DFDBA in treating intrabony defects. The present randomized controlled trial assessed for the first time the clinical and radiographic outcomes of I-PRF combined with DFDBA in the management of periodontal intrabony defects in patients with stage-III periodontitis. Clinical attachment level (CAL; primary outcome), periodontal probing depth (PPD), gingival recession depth (GRD), full-mouth plaque scores (FMPS), full-mouth bleeding scores (FMBS), radiographic linear defect depth (RLDD), and bone fill (secondary outcomes) were assessed at baseline, 3, 6, and 9 months post-surgically.

## Materials and methods

### Study registration and design

The current study was conducted in compliance with Helsinki Declaration for medical research involving human subjects as revised in 2013 as double-blind, parallel arms, and randomized controlled clinical trial, with 1:1 allocation ratio, to assess clinical and radiographic outcomes of I-PRF combined with DFDBA (I-PRF + DFDBA; test-group) versus DFDBA alone (DFDBA; control group) in surgical treatment of intrabony defects of stage-III periodontitis patients. The trial protocol was registered on www.clinicaltrials.gov on the 31st of March 2019 (NCT03900013), and the informed consents were approved by the Ethics Committee, Faculty of Dentistry, Cairo University on April 2019 (IRB:19|4|1).

### Population

**Fig. 1:**
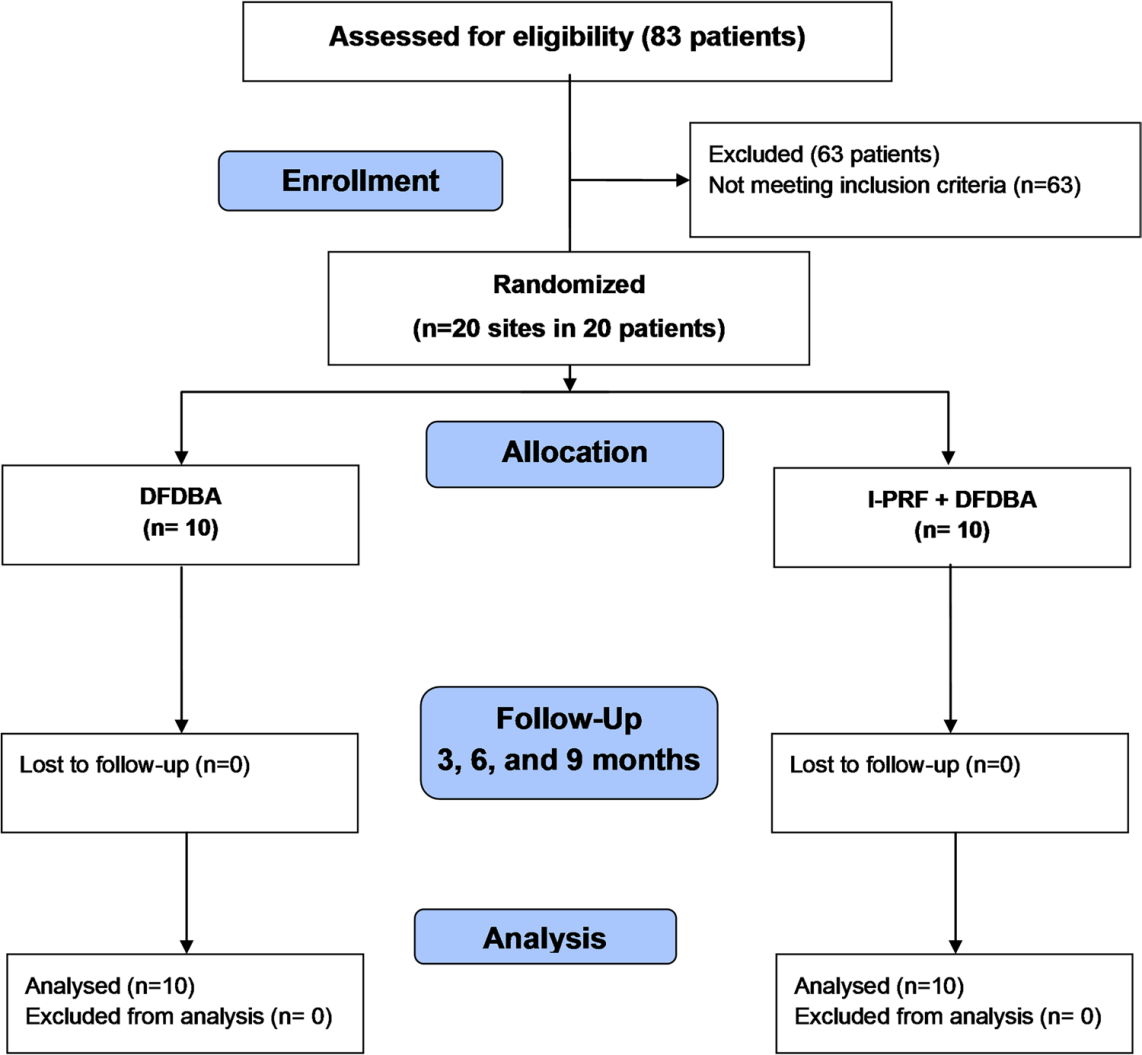
Flow diagram of patient recruitment and inclusion

Recruitment, operation, and follow-up of all participants were carried between June 2019 and July 2021 at the Department of Oral Medicine and Periodontology, Faculty of Dentistry, Cairo University, Egypt. Participants were enrolled through screening of patients at the Department of Periodontology, Cairo University, Egypt, personal referrals, and poster announcements. A total of 83 participants were assessed for eligibility, 63 are excluded for not meeting the inclusion criteria, and 20 participants (20 defects) were included (Fig. 1). All participants (age ≥ 18 years) were diagnosed with stage-III periodontitis, full-mouth plaque score (FMPS) and full-mouth bleeding scores (FMBS) ≤ 20% [14], PPD ≥ 6 mm, and CAL ≥ 5 mm, which persisted 6–8 weeks following non-surgical periodontal therapy [15], with ≥ 3 mm two- or three-walled intrabony defects detected radiographically. Patients with systemic conditions contradicting surgical intervention or affecting periodontal healing, including smokers [16, 17], diabetic patients [18], pregnant or lactating females [19], and patients with tooth mobility, furcation involvement, or active orthodontic therapy [20], were excluded.

### Sample size

Sample size calculation was conducted using a mean CAL difference of 1.1 mm, as the minimal clinically acceptable CAL difference, and a standard deviation of 0.74 mm [21]. Using a power of *β* = 80% and type I error *α* = 5% and based on two-tailed *t*-test, 8 defects were deemed necessary, which were increased to 10 defects per group to account for dropouts. Sample size calculation was performed, using G-Power software version 3.1 (Heinrich-Heine-Universität, Düsseldorf, Germany).

### Randomization and blinding

Participants were randomly assigned to either I-PRF + DFDBA- or DFDBA-group. Sequence generation was carried out using www.randomizer.org. Allocation was concealed in sequentially numbered opaque-sealed envelopes (MH). All participants were equally prepared for the surgical procedure by a single investigator (MA). Following open flap debridement (OFD), the study coordinator (KFE) assigned the participants to either I-PRF + DFDBA or DFDBA-group. Due to the type of interventions, the operator and participants could not be blinded. The outcome assessor and the biostatistician were blinded.

### Outcomes

CAL (primary outcome) was measured as the distance from the cemento-enamel junction (CEJ) to the base of periodontal pocket. PPD was determined as the distance from the base of pocket to the gingival margin. GRD was measured as the level from the gingival margin to the CEJ, while FMPS [22] and FMBS [23] were measured as previously described (all secondary outcomes). On the day of surgery, all baseline parameters were recorded. CAL, PPD, and GRD were measured at baseline, 3, 6, and 9 months post-operatively in mm, using UNC-15 periodontal probes and prefabricated customized acrylic stents with interproximal grooves to harbor the periodontal probe, for standardization and reproducibility of clinical measurements [24, 25]. FMPS and FMBS were measured at baseline and 9 months post-operatively. Changes in the recorded parameters were calculated through subtraction of 3, 6, and 9 months from baseline values, and percentage changes were determined through dividing values of changes by baseline values.

**Fig. 2:**
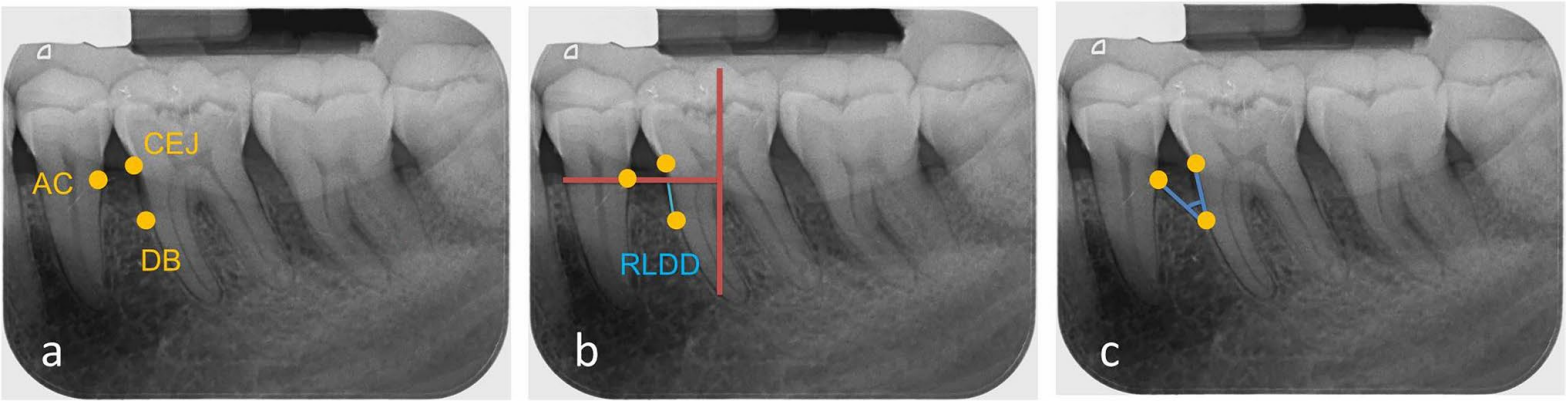
Intrabony defect radiographic measurements. **a** Reference point identification: cemento-enamel junction (CEJ), alveolar crest (AC), and defect base (DB). **b** Reference line identification (in red): vertical line corresponding to long axis and horizontal perpendicular line passing through AC and identifying radiographic linear defect depth (RLDD) in blue. **c** Radiographic angle connecting CEJ, DB, and AC

Individually customized bite blocks fabricated for each patient and parallel-angle technique were employed (Zhermack Zetaplus C-Silicone kit, Badia Polesine, Italy) using (XCP®) X-ray film holding system (Dentsply Sirona, Charlotte, USA). Periapical radiograph PSP sensor size two (Xios AE, Dentsply Sirona, New York, USA) and standardized exposure setting of 60 kVp, 8 mA, 0.7 mm, and 0.10 s (Heliodent Plus, Dentsply Sirona, PA, USA) were used. The defect angle was measured at baseline, as the angle formed between intersections of AC-DB and tooth long axis lines. RLDD was measured as the depth of the intrabony defect from the alveolar crest (AC) to the defect base (DB) as previously reported [26, 27] at baseline, 6 and 9 months postoperatively (Fig. 2). Calculation of bone fill in mm was done by a subtraction of follow-up from baseline RLDD values, and percentages of bone fill were expressed as the proportion of change to baseline RLDD [28].

### Calibration

Two blinded experienced investigators (WA and MN) obtained all parameters. Prior to study conduction, calibration was performed through comparing two measurements by the two investigators on the same participants (not included in the study) twice, one week apart, retrieving an intraexaminer agreement score > 0.85 for clinical outcomes and > 0.82 score for radiographic measurements.

### Interventions

#### Pre-operatively

Participants were provided with information about the intervention and asked to sign an informed consent. Phase I periodontal therapy was conducted through supra- and subgingival debridement, followed by instructions on oral hygiene performance, using toothbrushes and a twice daily use of 0.12% chlorhexidine HCL mouthwash for two weeks (Hexitol, ADCO Pharma Co, Cairo, Egypt) [15]. After 6–8 weeks, reevaluation was performed to confirm the necessity for surgical intervention through persistence of interproximal defects with PPD ≥ 6 mm, CAL ≥ 5 mm, and vertical intrabony defects ≥ 3 mm on periapical radiographs [29].

### Surgery

All surgical procedures were conducted by a single operator (MA). Following administration of local anesthesia (2% mepivacaine HCl with 1:20,000 levonordefrin, Alexandria Co. for Pharmaceuticals, Alexandria, Egypt), intrasulcular incisions were performed buccally and lingually/palatally on the affected tooth and extended one adjacent tooth mesially and distally, using 15c blades (TRINON Titanium GmbH, Augartenstraße, Karlsruhe, Germany). Following mucoperiosteal flaps’ elevation, thorough debridement was performed using ultrasonic scalers (Woodpecker Ultrasonic UDS-K Scaler, Zhengzhou, China) and mini-/after-five Gracey curettes (Hu-Friedy, Chicago, USA), until defects were clear from any granulation tissue [30], and the defect morphology was visually explored and recorded.

**Fig. 3:**
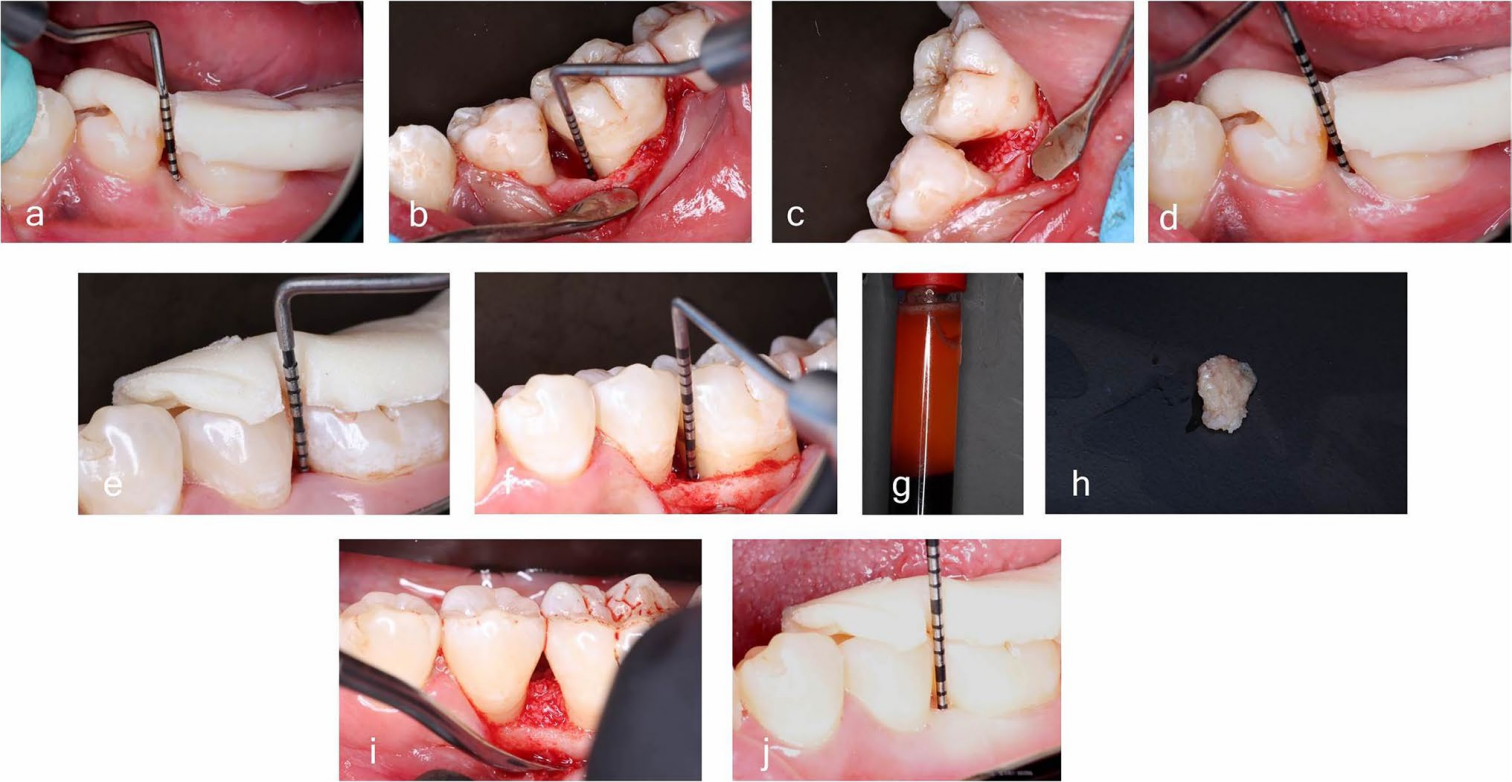
Clinical steps in representative cases of the control (**a–d**) and test (**e–j**) groups. Control group using DFDBA alone and test group using I-PRF/DFDBA. Control group: **a** 7 mm probing pocket depth using a prefabricated stent at baseline, **b** intrabony defect with vertical component of 4 mm at the mesial site of lower left first molar, **c** application of DFDBA in the defect, and **d** 3 mm proping pocket depth 9 months post-operatively. Test group: **e** 6 mm probing pocket depth at the mesial site of lower left first molar, **f** intrabony defect with vertical component of mm, **g** injectable-PRF, **h** combination of I-PRF with DFDBA, **i** application of combined I-PRF/DFDBA in the defect, and **j** 2 mm proping pocket depth 9 months post-operatively

In the control group, DFDBA graft material (AlloOss®; demineralized cortical particulates, ACE Surgical Supply Co., Brockton, MA, USA) was placed into the intrabony defect without overfilling. In the test group, 10 mm of fresh blood was withdrawn by venipuncture of the antecubital vein into a sterile 10 ml glass vacuum tube (Voma Med, Chongqing, China) without anticoagulant, and the tube immediately centrifuged (Digital Tabletop Centrifuge, rotor angle: 45° and a maximum radius of 10.6 cm, Velab, VE-4000, TX, USA) at a maximum relative centrifugal force (RCF-max) of 60 g (700 rpm) for 3 min at room temperature [31]. One milliliter of liquid I-PRF was collected using a sterile syringe [32, 33] and amalgamated with DFDBA at a proportion of 1:1 [21], till the I-PRF encapsulated the bone particles, before putting it into the intrabony defect. Finally, the flap was passively repositioned using interrupted 4–0 silk suture (ASSUT Medical, Pully-Lausanne, Switzerland; Fig. 3).

#### Post-operatively

One gram Augmentin (875 mg amoxicillin + 125 mg clavulanate potassium, GlaxosmithKline, Worthing, England) twice per day for 7 days and Ibuprofen 600 mg, three times daily for 3 days (Kahira Pharm Co., Cairo, Egypt) [34], were prescribed. Participants were instructed to avoid tooth brushing and flossing in the surgical area for two weeks and to rinse with 0.12% chlorhexidine HCL (Hexitol, ADCO Pharma) twice per day for 1 min [35]. Sutures were removed 14 days after the surgical procedure [34], and participants were advised to continue mechanical biofilm removal, using an ultra-soft toothbrush. Recalling of participants was done weekly during the first month then at 3, 6, and 9-months [36].

### Statistical analysis

Non-numerical descriptive data were presented as number (*n*) and percentage (%), using Chi-square test. Numerical data was reported as mean ± standard deviation or median with interquartile range. Kolmogorov–Smirnov test and Shapiro–Wilk test were used to explore the normality of data. For normally distributed data, independent *t*-test was used for intergroup comparison, while repeated measures ANOVA/Bonferroni post hoc correction (3 or more intervals) was employed for intragroup comparison between different time points. For non-normally distributed data, Mann-Whiney *U* test was used for intergroup comparison whilst Friedman test and post hoc Wilcoxon signed-rank test were used for intragroup comparison. A stepwise linear regression model used CAL after 9 months as the dependent variable, with study group, gender, age, number of defect walls, FMBS at baseline and 9 months, FMPS at baseline and 9 months, RLDD and radiographic defect angle at baseline, and bone fill at 9 months as independent variables. All tests were two-tailed, and *p* < 0.05 was statistically significant (SPSS for Windows, version 26, IBM, New York, USA).

## Results

### Baseline characteristics

This randomized, parallel-group clinical trial included a total of 20 intrabony defects in twenty participants (7 males and 13 females) with stage-III periodontitis. The periodontal intrabony defects were randomly assigned either into I-PRF + DFDBA-group (*n* = 10, test-group) or DFDBA-group (*n* = 10, control-group), with no drop-outs. The test group included 3 males and 7 females (mean age of 31.30 ± 4.79 years), while control group included 4 males and 6 females (mean age of 33.90 ± 6.44 years). Healing in all patients was uneventful. Regarding tooth distribution, the I-PRF + DFDBA-group involved three anteriors, two premolars, and five molars, while the DFDBA-group had four, one, and five teeth respectively (baseline characteristics in Table 1). Concerning defects’ morphology, the I-PRF + DFDBA-group comprised of 30% combined one–two walls, 20% combined two–three walls, 40% two-wall, and 10% three-wall defects, while the DFDBA-group included 20% combined one–two walls, 30% combined two–three walls, 30% two-wall, and 20% three-wall defects. The radiographic defect angle in the I-PRF + DFDBA-group was 35.62 ± 8.58° while 40.41 ± 8.24° in the DFDBA-group at baseline (*p* ≥ 0.05, Table 1).

**Table 1:** Baseline characteristics of age, gender, tooth location, intrabony defect morphology, and radiographic defect angle

	I-PRF + DFDBA (*n* = 10)	DFDBA (*n* = 10)	*p*-value
Age [years, mean ± SD]	31.30 ± 4.79	33.90 ± 6.44	0.319
Gender [*n* (%)]			
Male	3 (30%)	4 (40%)	0.639
Female	7 (70%)	6 (60%)	
Tooth location [*n* (%)]			
Anterior	3 (30%)	4 (40%)	0.788
Premolar	2 (20%)	1 (10%)	
Molar	5 (50%)	5 (50%)	
Intrabony defect morphology [*n* (%)]			
2 walls	4 (40%)	3 (30%)	0.831
3 walls	1 (10%)	2 (20%)	
Combined 1–2 walls	3 (30%)	2 (20%)	
Combined 2–3 walls	2 (20%)	3 (30%)	
Radiographic defect angle (degree)			
Baseline	35.62 ± 8.58	40.41 ± 8.24	0.219

### Clinical outcomes

Compared to baseline values, the I-PRF + DFDBA-group demonstrated significant CAL-gain of 2.20 ± 0.63 mm (38.48 ± 10.80%), 2.50 ± 0.53 mm (43.24 ± 6.67%), and 2.40 ± 0.70 mm (41.81 ± 11.31%, *p* < 0.05), while the DFDBA-group showed significant CAL-gain of 2.50 ± 1.08 mm (38.12 ± 7.92%), 2.70 ± 1.16 mm (41.55 ± 11.84%), and 2.50 ± 0.85 mm (40.77 ± 15.54%, *p* < 0.05) at 3, 6, and 9 months respectively, with insignificant intergroup differences (*p* ≥ 0.05, Table 2). Similarly, compared to baseline, a significant PPD-reduction was notable in the I-PRF + DFDBA-group of 2.50 ± 0.97 mm (36.23 ± 10.70%), 2.90 ± 0.74 mm (43.23 ± 8.74%), and 3.50 ± 1.18 mm (51.26 ± 11.50%, *p* < 0.05) and in the DFDBA-group of 2.40 ± 1.07 mm (35.98 ± 10.27%), 2.70 ± 0.67 mm (41.54 ± 7.69%), and 2.80 ± 0.42 mm (44.10 ± 9.82%, *p* < 0.05) at 3, 6, and 9 months respectively, with insignificant intergroup differences (*p* ≥ 0.05, Table 2). Compared to baseline, a significant change in GRD values of -0.80 ± 0.63 mm, − 0.50 ± 0.97 mm, and 0.10 ± 1.20 mm was evident in the I-PRF + DFDBA-group at 3, 6, and 9 months respectively (*p* < 0.05). In the DFDBA-group, GRD changes were − 1.40 ± 1.35 mm, − 1.20 ± 1.23 mm, and − 1.10 ± 1.45 mm at 3, 6, and 9 months respectively, with insignificant intergroup differences (*p* ≥ 0.05, Table 2). In the I-PRF + DFDBA-group, FMPS was 17.30 ± 2.21% and 12.90 ± 2.08% versus 17.70 ± 2.21% and 13.50 ± 1.58% in the DFDBA-group at baseline and 9 months respectively, with insignificant intergroup differences (*p* ≥ 0.05, Table 2). For FMBS, the I-PRF + DFDBA-group showed significant improvement from 18.30 ± 1.37% to 13.60 ± 2.12%, versus the DFDBA-group, which demonstrated 18.50 ± 1.27% and 14.10 ± 2.13% at baseline and 9 months respectively (*p* < 0.05), with insignificant intergroup differences (*p* ≥ 0.05, Table 2).

**Table 2:** Clinical outcomes

	I-PRF + DFDBA Mean (± SD)	DFDBA Mean (± SD)	Intergroup *p*-value
CAL (mm)			
Baseline	5.80 ± 0.92	6.50 ± 2.32	1.00
3 m	3.60 ± 0.97	4.00 ± 1.41	0.717
6 m	3.30 ± 0.67	3.80 ± 1.55	0.743
9 m	3.40 ± 0.97	4.00 ± 2.26	1.00
Intragroup *p*-value	< 0.001*	< 0.001*	
mm gain (3 m)	2.20 ± 0.63	2.50 ± 1.08	0.614
% gain (3 m)	38.48 ± 10.80	38.12 ± 7.92	0.934
mm gain (6 m)	2.50 ± 0.53	2.70 ± 1.16	0.837
% gain (6 m)	43.24 ± 6.67	41.55 ± 11.84	0.699
mm gain (9 m)	2.40 ± 0.70	2.50 ± 0.85	0.644
% gain (9 m)	41.81 ± 11.31	40.77 ± 15.54	0.866
PPD (mm)			
Baseline	6.70 ± 0.95	6.50 ± 1.08	0.472
3 m	4.20 ± 0.42^a^	4.10 ± 0.57^a^	0.689
6 m	3.80 ± 0.79^b^	3.80 ± 0.79^b^	1.00
9 m	3.20 ± 0.63^a^	3.70 ± 1.16^c^	0.325
Intragroup *p*-value	< 0.001*	< 0.001*	
mm reduction 3 m	2.50 ± 0.97	2.40 ± 1.07	0.522
mm reduction 6 m	2.90 ± 0.74	2.70 ± 0.67	0.534
mm reduction 9 m	3.50 ± 1.18	2.80 ± 0.42	0.095
Intragroup *p*-value	0.005*	0.319	
% reduction 3 m	36.23 ± 10.70	35.98 ± 10.27	0.959
% reduction 6 m	43.23 ± 8.74	41.54 ± 7.69	0.652
% reduction 9 m	51.26 ± 11.50^a^	44.10 ± 9.82	0.151
Intragroup *p*-value	0.003*	0.319	
GRD (mm)			
Recession at baseline	− 0.90 ± 0.99^a^	− 1.60 ± 2.01	0.337
Recession at 3 m	− 0.80 ± 0.63^b^	− 1.40 ± 1.35	0.219
Recession at 6 m	− 0.50 ± 0.97	− 1.20 ± 1.23	0.175
Recession at 9 m	0.10 ± 1.20^a,b^	− 1.10 ± 1.45	0.059
Intragroup *p*-value	0.006*	0.319	
FMPS (%)			
Baseline (%)	17.30 ± 2.21	17.70 ± 2.21	0.691
At 9 months (%)	12.90 ± 2.08	13.50 ± 1.58	0.477
Intragroup *p*-value	< 0.001*	< 0.001*	
FMBS (%)			
Baseline (%)	18.30 ± 1.37	18.50 ± 1.27	0.736
At 9 months (%)	13.60 ± 2.12	14.10 ± 2.13	0.605
Intragroup *p*-value	< 0.001*	< 0.001*	

^*^Statistically significant at *p* < 0.05. *CAL*, clinical attachment level; *PPD*, probing pocket depth; *GRD*, gingival recession depth; *FMPS*, full-mouth plaque scores; *FMBS*, full-mouth bleeding scores

### Radiographic outcomes

In the I-PRF + DFDBA-group, RLDD was 6.21 ± 1.22 mm at baseline and significantly decreased to 4.43 ± 0.80 mm at 6 months and 3.58 ± 0.66 mm at 9 months (*p* < 0.05). In the DFDBA-group, RLDD was further significantly decreased from 6.61 ± 2.07 mm at baseline, to 4.88 ± 1.46 mm and 3.89 ± 1.57 mm at 6 and 9 months respectively (*p* < 0.05), with insignificant intergroup differences (*p* ≥ 0.05, Table 3). The I-PRF + DFDBA-group showed radiographic bone fill of 1.78 ± 0.96 mm (27.83 ± 12.67%) after 6 months and 2.63 ± 0.95 mm (41.64 ± 10.43%) after 9 months (*p* < 0.05). Similarly, in the DFDBA-group, a significant radiographic bone fill of 1.73 ± 0.97 mm (25.52 ± 9.42%) and 2.72 ± 1.00 mm (41.35 ± 10.64%) was evident after 6 and 9 months respectively, with insignificant intergroup differences (*p* ≥ 0.05, Table 3).

**Table 3:** Radiographic outcomes

	I-PRF + DFDBA (*n* = 10)	DFDBA (*n* = 10)	Intergroup *p*-value
RLDD (mm)			
Baseline	6.21 ± 1.22^a^	6.61 ± 2.07^a^	0.605
6 m	4.43 ± 0.80^a^	4.88 ± 1.46^a^	0.403
9 m	3.58 ± 0.66^a^	3.89 ± 1.57^a^	0.572
Intragroup *p*-value	< 0.001*	< 0.001*	
Radiographic bone fill (mm)			
6 m	1.78 ± 0.96	1.73 ± 0.97	0.909
9 m	2.63 ± 0.95	2.72 ± 1.00	0.839
Intragroup *p*-value	< 0.001*	< 0.001*	
Radiographic bone fill (%)			
6 m	27.83 ± 12.67	25.52 ± 9.42	0.649
9 m	41.64 ± 10.43	41.35 ± 10.64	0.952
Intragroup *p*-value	< 0.001*	< 0.001*	

^*^Statistically significant *p* < 0.05. *RLDD*, radiographic linear defect depth

### Stepwise linear regression analysis

A significant direct relationship between CAL at 9 months and RLDD at baseline and a significant inverse relationship between CAL and bone fill and at nine months were evident (*p* < 0.05, Table 4).

**Table 4:** Stepwise linear regression analysis model for clinical attachment level at 9 months (β, regression coefficient; *SE*, standard error; *CI*, confidence interval; *FMBS*, full-mouth bleeding score; *FMPS*, full-mouth plaque score; *RLDD*, radiographic linear defect depth; significant differences are marked with asterisk; *: *p* < 0.05)

Variable	*Β*	95% CI for β		SE	*p*-value
Study group	− 0.035	− 1.094	1.024	0.459	0.941
Gender	0.173	− 0.776	1.123	0.412	0.689
Age	− 0.016	− 0.116	0.085	0.044	0.731
Number of walls	0.176	− 0.466	0.818	0.279	0.545
FMPS at baseline	0.119	− 0.178	0.415	0.128	0.382
FMPS at 9 months	− 0.091	− 0.478	0.296	0.168	0.601
FMBS at baseline	0.131	− 0.500	0.761	0.273	0.646
FMBS at 9 months	− 0.029	− 0.363	0.305	0.145	0.847
RLDD at baseline	1.483	0.962	2.003	0.226	0.001*
Radiographic angle at baseline	0.037	− 0.048	0.123	0.037	0.344
Radiographic bone fill at 9 months	− 1.703	− 2.5790	.827	0.380	0.002*

## Discussion

The persistence of intrabony defects, following non-surgical periodontal therapy, represents a risk factor for further disease progression [29]. Thus, a primary aim of individualized periodontal therapy remains to be a resolution of such defects with possible reinstitution of the lost tooth-investing and supporting structures [2, 37]. In recent years, autologous platelet concentrates were introduced as promising biological agents in the management of various periodontal defects with remarkable clinical results [38, 39], with PRF alone or in combination with bone replacement grafts reported to induce significant PPD-reduction and CAL-gain [40]. These results were primarily attributed to the PRF’s ability to enhance the periodontal wound healing events, providing three-dimensional fibrin scaffolds for cellular migration, adhesion, and differentiation, through its enclosed leukocytes and platelets, in addition to its continuous delivery of a multitude of crucial growth/differentiation factors into the wound site [41–43]. Additionally, the low-speed centrifugation concept, introduced to promote a higher and more uniform distribution of platelets and leukocytes within the PRF, resulted in enhanced PRF formulations, including the liquid I-PRF. Compared to conventional PRF, I-PRF was reported to demonstrate higher concentrations of growth/differentiation factors [31] and a more sustained release of these factors over a period of ten days [5, 6]. To our knowledge, the current randomized clinical trial is the first to explore the adjunctive effect of combining I-PRF with DFDBA in the surgical treatment of intrabony periodontal defects.

The amalgamation of platelet concentrates with bone grafts, in addition to enhancing the grafts’ clinical handling properties, would entrap platelets and neutrophils and release essential growth/differentiation factors in the healing periodontal site [44, 45]. Indeed, similar to earlier investigations combining PRP [46] and PRF [47] with DFDBA, or PRF with demineralized bovine bone matrix (DBBM) [48] in the management of intrabony defects, in the current study, I-PRF + DFDBA as well as DFDBA alone exhibited significant CAL-gain, PPD-reduction, and radiographic bone fill, with no significant differences observed between them. Similarly, the addition of a PRF membrane to bioactive glass did not enhance periodontal clinical parameters in terms of PPD-reduction and CAL-gain compared to bioactive glass alone after 9 months, although more significant bone fill was evident in the intrabony defects receiving the combined treatment after 6 and 9 months [49]. Yet, PRF used in the form of membranes with DFDBA [21] or bioactive glass [50] demonstrated significantly enhanced CAL-gain, PPD-reduction, and bone fill compared with DFDBA or bioactive glass alone, allowing for the plausible assumption that, in contrast to the above-mentioned results, the growth/differentiation factors laden PRF membranes could have provide a short-term compartmentalization effect that could augment their periodontal reparative/regenerative effects. Comparable to previous studies, demonstrating a favorable effect of PRF on soft tissue healing attributes [48, 50, 51], in the present study, I-PRF + DFDBA-group exhibited an enhanced yet non-significant, reduction in gingival recession parameters, underlying the earlier reported favorable effects of the fibrin content of I-PRF, exerting cellular adhesive and migration promoting functions, stabilizing the surgical flap, enriching the area with a multitude of essential growth/differentiation factors, and thereby enhancing angiogenesis, epithelialization, and soft tissue wound healing [52].

Although it was demonstrated that platelet concentrates resulting from the low-speed centrifugation concept release a higher amount of growth/differentiation over time [53], compared to A-PRF, the total number of leukocytes, platelets, and growth/differentiation factors could have been significantly lower in the obtained I-PRF, owing to its lesser volume [6]. This could explain the observed absence of a significant synergistic effect of I-PRF/DFDBA amalgamation on the examined periodontal parameters. A further explanation for the absence of an additional effect, similar to earlier results on the combination of EMD with DFDBA in the treatment of intrabony defects [4], could be that the biological effects of I-PRF have been masked in the amalgamate by the outstanding osteoconductive properties of the DFDBA, harboring itself an array of growth/differentiation factors in higher amounts (BMP-2, -4, and -7; TGF-b1, VEGF, FGF-a, and IGF-I) pivotal for various early and especially late stages of periodontal wound healing [54–56]. Finally, the regression model did not show associations between age, gender, number of defect walls, radiographic angle, FMPS, and FMBS at baseline or follow-ups with CAL at 9 months. However, a significant direct association between RLDD at baseline and CAL was evident. Moreover, a significant inverse relationship between bone fill gain and CAL at nine months was evident, as bone gain measured radiographically is translated into a reduced attachment loss clinically.

Still, the current trial’s results should be interpreted in context of its limitations. First, the inclusion of intrabony defects with different morphologies, although randomly distributed could have affected the observed effects. A subgrouping according to the defects’ morphology, although being more informative, would have led to substantial decrease in the trial’s power. Second, the preparation of I-PRF necessitates collection of patient’s own blood. Consequently, patients who are afraid of blood sampling repelled to participate in the current trail. Third, the present study did not use the newly developed horizontal centrifugation protocol [6], which could have elevated the number of platelets and leucocytes in the I-PRF, with a more even platelet distribution. Fourth, although minimally invasive surgical techniques are currently recommended in regenerative therapeutic approaches of intrabony defects [57], these procedures were not applied in the current study due to the presence of deep intrabony defects, involving three or four sides of the root of the affected teeth, that often necessitated more extension of the flap for sufficient visibility for instrumentation and efficient debridement of the intrabony defects and the affected root surfaces [58]. Thus, instead, the standard OFD was employed. Finally, as the included patients stemmed from lower socio-economical levels solely interested in a symptomatic therapy, it was not feasible to reliably include patient-reported outcomes (as self-reported pain scores) in the current investigation.

Within the limitations of current trial, it can be concluded that both treatment modalities (I-PRF + DFDBA and DFDBA alone) resulted in significant improvement in clinical and radiographic parameters 9 months post-surgically. Apart from an observed improvement in gingival recession, combining I-PRF with DFDBA did not appear to significantly augment the DFDBA’s therapeutic outcomes. Further longitudinal clinical and histological studies with larger sample sizes are needed to fully explore the regenerative potential of I-PRF in combination with DFDBA and its efficacy in the treatment of intrabony periodontal defects.

## Data Availability

Data available on request due to privacy/ethical restrictions.
